# Bisphenols and Leydig Cell Development and Function

**DOI:** 10.3389/fendo.2020.00447

**Published:** 2020-07-31

**Authors:** Xiaoheng Li, Zina Wen, Yiyan Wang, Jiaying Mo, Ying Zhong, Ren-Shan Ge

**Affiliations:** ^1^Department of Obstetrics and Gynecology, The Second Affiliated Hospital and Yuying Children's Hospital, Wenzhou Medical University, Wenzhou, China; ^2^Chengdu Xi'nan Gynecology Hospital, Chengdu, China

**Keywords:** bisphenol, bisphenol analogs, Leydig cells, steroids, reproductive function

## Abstract

Bisphenol A (BPA) is a ubiquitous environmental pollutant, mainly from the production and use of plastics and the degradation of wastes related to industrial plastics. Evidence from laboratory animal and human studies supports the view that BPA has an endocrine disrupting effect on Leydig cell development and function. To better understand the adverse effects of BPA, we reviewed its role and mechanism by analyzing rodent data *in vivo* and *in vitro* and human epidemiological evidence. BPA has estrogen and anti-androgen effects, thereby destroying the development and function of Leydig cells and causing related reproductive diseases such as testicular dysgenesis syndrome, delayed puberty, and subfertility/infertility. Due to the limitation of BPA production, the increased use of BPA analogs has also attracted attention to these new chemicals. They may share actions and mechanisms similar to or different from BPA.

## Introduction

Leydig cells (LCs) are a group of cells specifically located in the interstitium of the testis [see review (1)]. They secrete two important hormones: testosterone (T, androgen), which is an androgen, and insulin-like 3 (INSL3) [see review (2)]. There are at least two generations of LCs, namely fetal LCs (FLCs) and adult LCs (ALCs) (2). These two generations of LCs have different development processes and different functions (2). In fetuses, T and metabolically activated dihydrotestosterone (DHT) from T by 5α-reductase is essential for the development of the male reproductive tract (3). Failure to synthesize T may cause abnormalities in the male reproductive tract, such as hypospadias and small penis [see review (4)]. Androgens are also essential for testis descent (4). INSL3 binds to its receptor in the gubernaculum and pulls the testis from the kidney position to the lower part of abdomen (5). *Insl3* knockout in mice leads to cryptorchidism, indicating that it is important for testis descent (6, 7). Therefore, defects in FLCs may cause the fetal part of Testicular Dysgenesis Syndrome (TDS) (8). TDS was coined to refer to diseases such as cryptorchidism and hypospadias in neonates and testicular cancer, as well as decreased fertility in men with common fetal causes (9). Although the exact cause is unclear, the high incidence of male reproductive tract defects in male neonates has brought significant attention to children's health (10, 11). In adults, T is essential for the onset of puberty, the maintenance of secondary sexual characteristics, the promotion of spermatogenesis, and the promotion of muscle health (4). INSL3 is essential for regulating bone metabolism in adult males (12) and acts as an anti-apoptotic factor against germ cell apoptosis (13).

**Figure 1:**
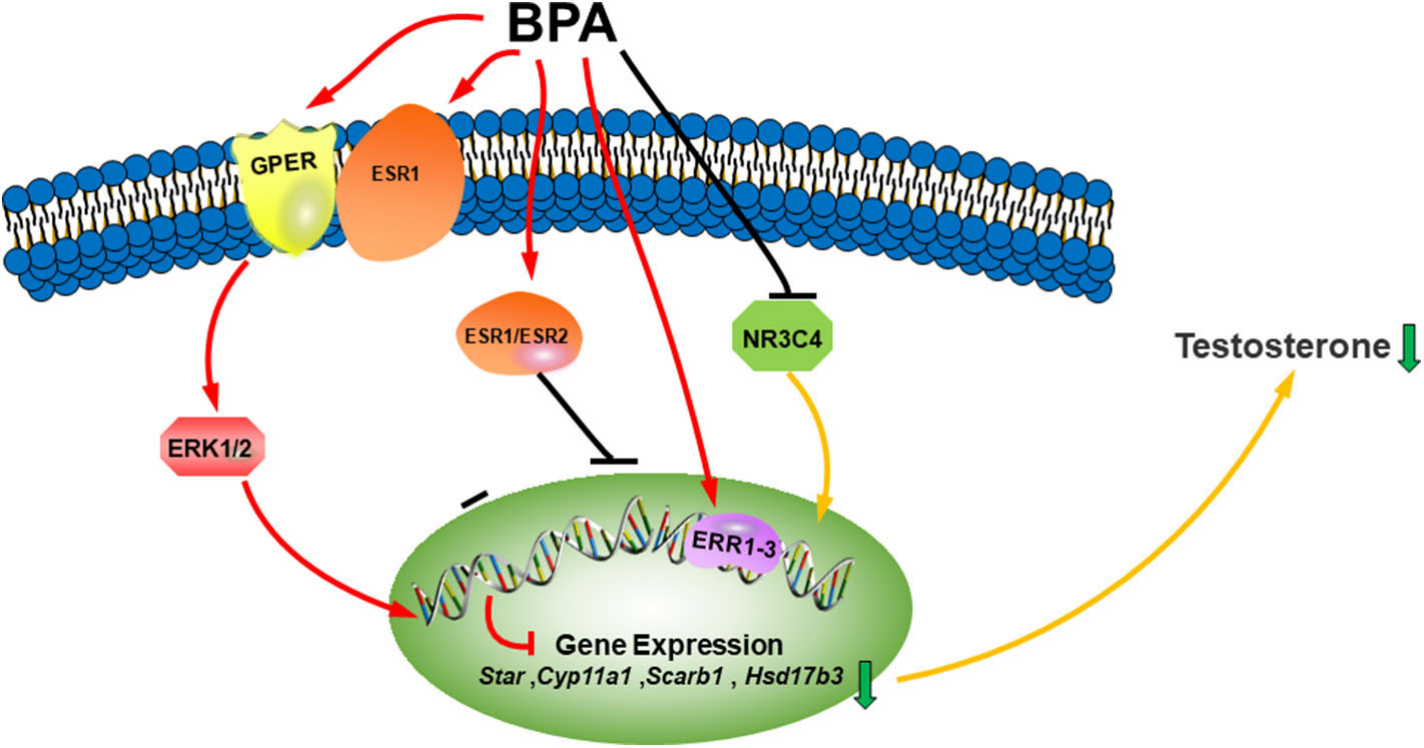
Illustration of BPA on LC development. Bisphenol A (BPA) or its analogs can bind both estrogen receptors (ESR1 and ESR2) and estrogen-related receptors (ERR1–3), which blocks LC gene expression, binds to androgen receptor (NR3C4) as an antagonist to block the activation of LC genes. BPA can also bind membrane G-coupled receptor (GPER) or ESR1, which activates ERK1/2 pathway to inhibit the differentiation of Leydig cells. The combined consequence of BPA action leads to lower testosterone synthesis.

There is increasing evidence that environmental pollutants can cause TDS, androgen deficiency, and infertility. A group of highly studied environmental chemicals comprises bisphenol A [2,2-bis (4-hydroxyphenyl) propane, BPA, Figure 1] and related compounds, such as bisphenol AF, AP, B, C, F, H, S, Z, and other similar chemicals (Table 1).

**Table 1:** Bisphenol analogs and their structures.

**Bisphenols**	**Abbreviation**	**CAS No**.	**MW**	**Structure**
Bisphenol A	BPA	80-05-7	228.28	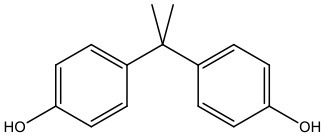
Bisphenol AF	BPAF	1478-61-1	336.23	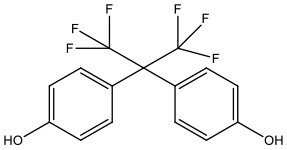
Bisphenol AP	BPAP	1571-75-1	290.36	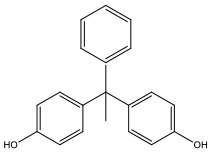
Bisphenol B	BPB	77-40-7	242.31	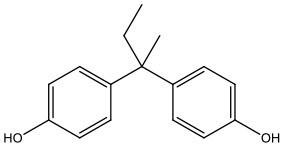
Bisphenol C	BPC	14868-03-2	281.13	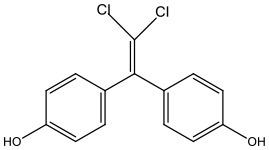
Bisphenol E	BPE	2081-08-5	12.24	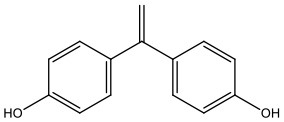
Bisphenol F	BPF	620-92-8	200.24	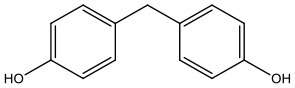
Bisphenol FL	BPFL	3236-71-3	350.41	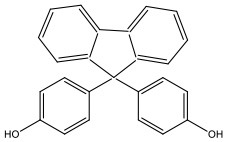
Bisphenol H	BPH	24038-68-4	380.48	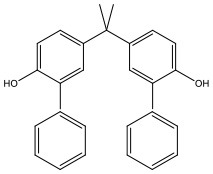
Bisphenol P	BPP	2167-51-3	346.50	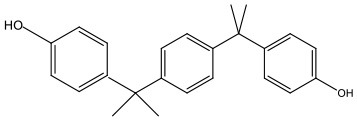
Bisphenol S	BPS	201-250-5	250.27	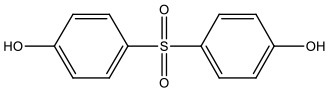
Bisphenol Z	BPZ	843-55-0	268.35	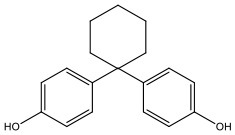
4,4′-Thiodiphenol	TDP	2664-63-3	218.27	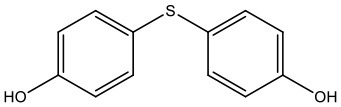
Tetramethyl bisphenol A	TMBPA	5613-46-7	284.39	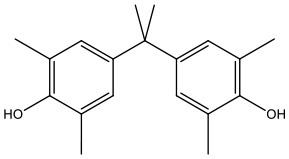

BPA is widely used in our industrial and consumer products and seriously pollutes our environment. BPA was first synthesized in 1891. Since then, BPA has been widely used in various products and applications as a common ingredient in plastic manufacturing. Plastics containing BPA are used to make children's toys, food containers, water bottles, medical equipment, and other durable materials (14–16). Many countries and regions are synthesizing BPA, including the United States, China, and European countries (16–19). Plastics are widely used in our consumer products and have changed our lifestyles, including the environment (20, 21). The widespread use of BPA-containing plastics has prompted BPA to spread in the environment. Therefore, BPA is ubiquitous in the environment, including air, drinking water, water systems, sewage sludge, soil, house dust, and food (16, 22). Humans are exposed to BPA mainly through food intake, dust, and skin contact (14, 15). BPA exposure through water and food is considered to be the main source (16, 22). Surveys indicate that 90% of urine samples in the general population of the United States can detect BPA levels (14, 17). The average urine BPA concentration in American people is about 2.5–10.95 ng/ml (14, 17). BPA can also penetrate the placenta and enter the fetal circulation. The average level of BPA in pregnant women's plasma is 0.3–18.9 ng/ml, and the average level of BPA in fetal plasma is 0.2–9.2 ng/ml (23, 24), and the level of BPA in placental tissue is 1.0–104.9 ng/g. BPA can enter breast milk, and the BPA level in breast milk is 0.28–0.97 ng/ml (23, 24). After ingestion through the oral route, BPA rapidly combines with blood proteins, and the concentration of free BPA in the blood is about 1 ng/ml (15).

There is increasing evidence that BPA is associated with the occurrence of reproductive toxicity (25, 26) and other health problems such as diabetes (27), neurotoxicity (28–30), immunotoxicity (31), and cancer (32–34).

BPA is classified as an endocrine disruptor that mainly mimics the effects of estrogen and disrupts the synthesis of male androgens (35–37). BPA is one of the most studied endocrine disrupting compounds. The toxicological effects of BPA may cause TDS (38) and other reproductive toxicities. The relationship between BPA and TDS and other reproductive effects has been well-studied in human epidemiology (18, 19, 39, 40). Due to the reproductive toxicity of BPA, some new BPA analogs, such as bisphenol AF, AP, B, C, F, H, S, and Z, were introduced into the market (Table 1) (41–43). These new compounds have received little attention. Many data on BPA reproductive toxicity have been collected from mice and rats. In this review, we mainly discuss the effects of BPA and its analogs on the development and function of LCs.

## Action of BPA

### Estrogen Receptors

The classic mechanism of estrogens requires them to bind to estrogen receptor (ESR), a type of nuclear receptor (44). There are two subtypes of ESR, namely ESR1 and ESR2 (45, 46). Estrogen binds to ESR to form a nuclear ESR dimer that binds to the ligand. This dimer binds to the DNA sequence (GGTCACAGTGACC) and is called an estrogen response element (ERE) in the target gene promoter to induce ESR transactivation (44). ESRs bind to the same sets of ERE in the target genes (47). When different isoforms exist in the same cell, the ESR bound to the ligand can form homodimers or heterodimers.

In addition to the genomic pathway of ESR, cytoplasmic/membrane-bound ESR interacts with many other proteins to mediate the activation of several kinase pathways that are hormone-dependent (48).

Estrogen-related receptors α, β, and γ (ERRα, ERRβ, and ERRγ, also known as ERR1–3) are another subfamily of orphan nuclear receptors with sequence similarity to ESR1 (49). However, 17β-estradiol (E2) is not its natural ligand, and ERR has constitutive activity (50). ERRs contain a DNA binding domain with two highly conserved zinc finger motifs in a specific DNA binding element (TCAAGGTCA, called ERRE). ERR and ERRE are combined into monomers or homodimers or heterodimers with coactivators (51). In addition to ERRE, ERR can also be bound to ERE. ESR1, but not ESR2, can also be combined with ERRE (52), so ESR1 and ERRs will affect each other.

In addition, estrogen can bind to G protein-coupled membrane estrogen receptor (GPER, also known as GPR30), which is a member of the G protein receptor superfamily. This receptor mediates the rapid signaling of estrogen. After activation, estrogen can induce ERK1/2 activation by releasing HB-EGF through transactivation of EGFR (53). GPER works through a pertussis toxin-sensitive pathway that depends on Gβγ (53). Then, GPER activation through Gαs protein activation (54) stimulates adenylate cyclase, increases cAMP, and weakens the EGFR-MAPK signaling axis (55). The activation of cAMP further leads to the activation of PKA-CREB signal (56, 57) and the transcriptional activation of CREB. GPER also activates other signaling, including PI3K (58), PKC (59), and calcium (60).

### Estrogen Receptors in LCs

In rodents, there are two generations of LCs: namely, FLCs and ALCs (2). The two generations of LCs have different development trajectories and functions (2, 61). The first generation of FLCs was found in fetal age (GD) 12 of mice, GD14 in rats, and fetal testes of human around gestational age (GW) 6 (62, 63). After birth, FLCs involute, and a few FLCs persist in the adult testes (64, 65).

The second-generation ALCs begin to develop around the 9th day after birth (PND) in rats, transit to progenitor LCs in PND21 (pre-pubertal period), develop into immature LCs during PND28-35, and finally mature to ALCs around PND56 (66).

ESRs, ERRs, and GER are differently expressed in LCs during the development, depending on two generations of LCs and species. ESR1 has been detected in mouse (67) and rat (68) FLCs, as well as mouse (69) and rat (70) ALCs. ESR2 was also found in mouse and rat (71) FLCs as well as mouse and rat (72) ALCs. It has been shown that the GPER level of rat LC is higher (73). In human fetal testes, ESR1 and ESR2 are located in FLCs (74, 75). Human LCs also have low levels of ESR1 and ESR2 and high levels of GPER (76–78). All three ERRs are found in mouse tumor LCs (79). In ESR1 knockout mice, ALCs are hypertrophic and serum T levels are elevated (80, 81). However, the ESR2 knockout mice did not change, but the average cell volume of ALC decreased (81).

### The Action of BPA and Its Analogs via Estrogen and Estrogen-Related Receptors in LCs

Both FLCs and ALCs mainly synthesize T from steroid cholesterol. High-density lipoprotein transport through scavenger receptor class B member 1 (SCARB1) contributes to the formation of most cholesterol in LCs (82, 83). Under the stimulation of luteinizing hormone (LH) or human chorionic gonadotropin (hCG) by binding to LH receptor (LHCGR) on the surface of LCs, adenylate cyclase is activated to increase intracellular adenosine 3′,5′cyclic monophosphate (cAMP) levels, triggering protein kinase A signaling (84). Then, the expression and phosphorylation of steroidogenic acute regulatory protein (StAR) is activated (85, 86) and, together with the translocation protein (TSPO) (87), they transport cholesterol to the mitochondrial inner membrane. In this organelle, there is a complex of P450 cholesterol side chain cleavage enzyme (CYP11A1), which catalyzes the production of pregnenolone by cholesterol (88). Pregnenolone diffuses from the mitochondria to the smooth endoplasmic reticulum, where 3β-hydroxysteroid dehydrogenase (HSD3B), 17α-hydroxylase/17,20-lyase (CYP17A1), and 17β- hydroxysteroid dehydrogenase 3 (HSD17B3) catalyzes a chain-reaction to generate T (89).

INSL3 is encoded by *Insl3* in LCs and is secreted into the circulatory system (6, 90). *Insl3* is also only expressed by two types of LCs. *Insl3* encodes a G protein, a leucine-rich repeat sequence GPCR 8 (also known as relaxin family peptide receptor 2, RXFP2). Knockdown of INSL3 or RXFP2 resulted in failure of testis descent (90–92), indicating that INSL3 is critical for testis descent. INSL3 constitutive expression depends only on LC number, but also on differentiation status. INSL3 is different from T. When T synthesis is low, it is restored to normal levels by supplementing LH levels (93). Because INSL3 is specifically expressed by LCs, INSL3 is a powerful sensitive biomarker that is affected by environmental endocrine disruptors even when exposed to them during pregnancy (94).

The detailed mechanisms of BPA and its analogs to interfere with LC functions has been reviewed (95). When ESR1 was used to compare the estrogen potency of BPA with E2 through the endogenous estrogen regulatory gene in human MCF7 cells, the potency of BPA was found to be four to six orders of magnitude lower than E2 (96). Some of these studies have shown that the BPA analog BPS has lower estrogen potency than E2 when measured in nuclear receptor models. However, BPS has the same or higher estrogen potency as E2 by binding membrane ESR [see review (95)].

In mouse MLTC-1 tumor LCs, BPA, and E2 have similar potency and can inhibit LH-stimulated cAMP production with <0.7 nM after 1 h, which may be caused by GPER (97).

Mouse tumor LCs express ERRs (79). BPA significantly binds to human ERR3, with an IC_50_ value of 13.1 nM, and binds to the ERR3 receptor cavity, and its two OH groups form a hydrogen bond; one forms a hydrogen bond with Glu275 and Arg316, and the other binds to Asn346 (98). BPA and ERR3 effectively bind as antagonists (28), and may inhibit cAMP-induced *Star* promoter activation by inhibiting the transcriptional activity of Nur77 (99). These results indicate that BPA works by binding different receptors, depending on the concentration. High concentrations of BPA mainly target ESR1, while low concentrations of BPA mainly target GPER and ERR3.

### Direct Inhibition on LC Steroidogenic Enzymes

Besides the receptor-mediated actions, BPA also directly interferes with androgen synthesis. The direct effects of BPA on rat and human T synthetic enzymes, including CYP17A1, HSD3B, and HSD17B3, were evaluated using testis microsomes. BPA directly inhibited rat and human CYP17A1, HSD3B, and HSD17B3 enzyme activities. IC_50_ values of BPA for rat and human testicular HSD3B were about 27 and 8 μM; IC_50_ values for rat and human CYP17A1 were about 65 and 19 μM, and BPA inhibited both rat and human HSD17B3 around 100 μM (100). Adult rat LCs also express both HSD11B1 and HSD11B2 (101), behaving in oxidative inactivation of cortisol or corticosterone, which can suppress androgen synthesis (102). BPA inhibited human HSD11B1, with an IC_50_ of about 15 μM and rat enzyme with IC_50_ of about 19 μM. BPA also weakly inhibited both human and rat HSD11B2 with IC_50_ values about 100 or over 100 μM (103). These results indicate that BPA directly inhibits steroidogenic enzyme activities at the higher concentrations.

### Other Mechanisms of BPA

Studies using *Nr3c4* (androgen receptor) knockout mice (104, 105) and Tfm mice (106, 107) showed that androgen is very critical for LC development. Knockout of *Nr3c4* in Sertoli cells, LCs, and peritubular myoid cells (104, 105) also caused the delay of ALC development. BPA might act as antiandrogen via blocking the activation of NR3C4. Lee et al. used a yeast detection system for the antiandrogenic effects of BPA and found that BPA antagonized DHT binding at 50 nM (108).

BPA-induced reactive oxygen species (ROS) generation has also been proposed for BPA-mediated suppression of T synthesis in LCs. ROS has been shown to disrupt LC steroidogenesis (109, 110). BPA was orally administered to adult male rats at 0.005, 0.5, 50, and 500 μg/kg/day for 45 days, and it significantly increased testicular ROS levels, suggesting that BPA-induced ROS might also be involved in its inhibition of T synthesis in LCs (111). Rats were administered BPA via gavage at 10 mg/kg/day BPA for 14 days, and it lowered T levels and decreased testis weight and inhibited antioxidants (such as SOD2 and catalase) and co-treatment with an antioxidant (lipoic acid) was able to reverse it (112). Male adult rats were administrated via gavage of 200 mg/kg BPA for 4 weeks, and it inhibited serum LH and T levels after decreasing SOD, GPx, and GSH and increasing ROS generation and antioxidants can attenuate BPA-induced inhibition (113). Adult male Wistar albino rats (aged 3 months) were gavaged with 50, 500, and 1,000 μg/kg BPA and/or vitamin E (40 mg/kg) for 3 months, and BPA significantly lowered T levels, testis weights, and sperm count, and vitamin E could attenuate it (114). These results indicate that BPA at high or very high doses also increases ROS levels.

## Animal Studies

### Effect of *in utero* BPA Exposure on Male Reproductive Tract Development

Reports on the effects of *in utero* BPA exposure on T production and male reproductive tract development are conflicting. This difference may be due to the dosage, developmental period, and species. Pregnant CD mice orally exposed to 50 μg/kg BW/day BPA from GD16 to 18 and F2 male pups had an increase in AGD on PND3 (115) (Table 2).

**Table 2:** Bisphenol A (BPA) and animal studies.

**Species**	**Regimen**	**Outcome**	**References**
***In utero*** **exposure on FLC development and function**			
CD mouse	po 50 μg/kg/day from GD16 to 18	Increase of AGD and decrease of epididymal weight without affecting testicular weights at PND3	(115)
SD rat	po 4–400 mg/kg/day from GD12 to 21	Reduction of serum T levels and expression of *Lhcgr, Insl3*, and *Hsd17b3* and FLC proliferation at 40 or 400 mg/kg	(26)
SD rat	s.c. 0.002–400 mg/kg/day from GD11 to 20	Reduction of expression of *Scarb1, Star, Cyp11a1, Cyp17a1*, and *Svs5* at 400 mg/kg	(116)
SD rat	s.c. 02–400 mg/kg/day from GD11 to 20	Reduction of expression of *Star* at 400 mg/kg	(117)
***In utero*** **exposure on postnatal LC development and function**			
SD rat	po 4 or 40 mg/kg/day form GD6 to PND20	Effect on at PND21 and inconclusive effect on T synthesis	(118)
LE rat	po 2–200 μg/kg/day from GD7 to PND18	No effect on AGD examined at PND2 and nipple retention at PND14	(119)
CD mouse	po 50 μg/kg/day from GD16 to 18	Decrease in epididymal weight without affecting testicular weights at PND21 and 60	(115)
CD mouse	po 2, 20 μg/kg/day from GD11 to 17	Reduction of relative testicular weights at PND56 and 84 without affecting serum T levels	(120)
SD rat	po 0.0025–250 mg/kg/day from GD6 to PND90	Reduction of testis/epididymis weights only at 250 mg/kg	(121)
LE Rat	po 2.5–25 μg/kg/day from GD12 to PND21	Increase of LC number and reduction of LHCGR and HSD17B3 and T secretion at PND90	(122)
**Neonatal exposure on postnatal LC development and function**			
SD rat	s.c. 0.002–97 mg/kg/day from PND0-9	No effect of preputial separation, T levels, and fertility rate on PND10 and PND150	(123)
LE rat	po 2.4 μg/kg/day from PND21 to 35	Reduction of serum LH and T levels	(124)
**Adult exposure on postnatal LC development and function**			
Swiss mouse	po 5–100 μ/kg BW/day from PND21 to 35	Reduction of absolute testis weights, seminal vesicle weight and sperm counts and fertility rate	(125)
SD rat	po from PND21 for 56 days	Reduction of free T levels without affecting LH levels	(126)
Wistar rat	s.c. 20–200 mg/kg BW BPA from PND21 for 42 days	Inhibition of plasma T and LH levels and down-regulation of *Cyp11a1* and *Scarb1*	(127)
SD rat	s.c. 1 mg/kg BW BPA at adulthood for 14 days	Decrease in plasma T level and increase in LH level	(128)
SD rat	po 10 mg/kg BW BPA at adulthood for 14 days	Reduction of serum T levels and testis weight	(112)
SD rat	po 0.005–500 μg/kg BW BPA at adulthood for 45 days	the testis as well as HSD3B1, HSD17B3, and StAR protein levels and T levels	(111)

*AGD, anogenital distance; GS, gestational day; LH, luteinizing hormone; PND, postnatal day; po, gavage; s.c., subcutaneous; T, testosterone*.

However, other studies have shown that BPA inhibits T synthesis in fetal testes. Oral administration of BPA from GD1 to GD22 to pregnant rats inhibited T production in neonates (129). Pregnant Sprague Dawley rats were administered 4, 40, and 400 mg/kg BW BPA via gavage daily from GD12 to 21, and BPA dose-dependently reduced serum T levels and down-regulated the expression of *Insl3* and *Hsd17b3* and their proteins at 40 and 400 mg/kg and that of *Lhcgr, Cyp11a1*, and *Cyp17a1* and their proteins at 400 mg/kg (26). BPA inhibited FLC proliferation at 400 mg/kg (26). Pregnant Sprague Dawley rats were administered 0.002, 0.02, 0.5, 50, or 400 mg/kg BW or 0.001, 0.01, 0.1, 1, or 10 μg/kg BW 17α-ethynyl estradiol (EE, as the positive control of ESR1 agonist) daily s.c. from GD11 to GD20. Gene microarray analysis in GD20 fetal testes revealed that BPA at 400 mg/kg and EE at 10 μg/kg significantly down-regulated the expression of FLC genes, including *Scarb1, Star, Cyp11a1, Cyp17a1*, and *Svs5* (116), and they had similar down-regulation patterns, suggesting that BPA exerts ESR1-mediated inhibition of FLC function (116). High doses of BPA exert similar effects to E_2_. Horstman et al. also exposed pregnant Sprague Dawley rats to 0.001 or 0.1 μg/kg BW/day EE or 0.02, 0.5, and 400 mg/kg/day BPA via s.c. from GD11 to GD20 and found that the highest concentration of EE and BPA down-regulated the expression of *Star* gene and proteins (117) (Table 2). These studies indicate that BPA may show different actions at low and high doses and it may mainly bind to ESR1 to take action at the high doses.

### Effects of *in utero* BPA Exposure on Postnatal Male Reproduction

There are also conflicting reports about the effects of *in utero* BPA exposure on the production of T after birth. This difference may also be due to dose, duration of treatment, and species. Pregnant rats were orally administered with 4 or 40 mg/kg BW/day BPA from GD6 to PND20, and BPA did not affect AGD in PND21 male offspring. This study cannot conclude the inhibitory effect of BPA on T secretion (118). From GD7 to PND18, pregnant Long Evans rats were administered doses of 2, 20, and 200 μg/kg BW/day, which had no effect on AGD at PND2 and nipple retention at PND14 in male offspring, suggesting that low doses of BPA cannot cause TDS (119). Pregnant mice were exposed to 50 μg/kg BW/day BPA from GD16 to GD18, which increased AGD and prostate size and decreased epididymal weight without affecting testicular weight at PND3, 21, and 60 (115) (Table 2).

Pregnant CD-1 mice who were administered low doses of BPA (2 and 20 μg/kg/day) via gavage of from GD11 to GD17 had significantly lower relative testicular weight compared to 8 and 12-week-old male mice without affecting serum T levels (120). Sprague Dawley pregnant rats were given 0.006, 0.025, 0.25, 2.5, 25, and 250 mg/kg BW/day by oral administration from GD6 to GD21, and their male pups were directly administered via gavage of the same doses of BPA from PND1 to PND90. BPA only suppressed the weight of testes and epididymis at a dose of 250 mg/kg (121) (Table 2).

However, when pregnant CD-1 mice were given 0.1, 1, or 10 mg/kg BPA BW/day by gavage and another plasticizer bis (2-ethylhexyl)-phthalate (DEHP) from GD1 to GD21, and further, in the weaning period (PND1-21), the mixture down-regulated the *Star* expression and reduced sperm count in epididymis at PND42 (130). This effect may be confused by the addition of DEHP. In pregnant Long-Evans rats gavaged with 2.5–25 μg/kg BW/day from GD12 to PND21, BPA stimulated LC proliferation during prepuberty and increased the number of LCs at PND90, but down-regulated LHCGR and HSD17B3 and decreased T secretion by LCs (122) (Table 2). These different actions of BPA might be due to the doses of BPA.

### Effects of Neonatal and Prepubertal BPA Exposure on Postnatal Male Reproduction

There are also conflicting reports on the effects of BPA exposure on postnatal T production and reproduction. This difference may also be due to dose, duration of treatment, and species. Male Sprague Dawley rats were daily s.c. administered 0.002–97 mg/kg BW BPA or 0.9 mg/kg BW E_2_ from PND0 to PND9, and BPA did not affect preputial separation (an androgen-dependent process), T levels, and fertility rate on PND10 and PND150, while E_2_ inhibited these parameters (123). However, Long Even rats were orally exposed to 2.4 μg/kg BW/day BPA from PND21 to PND35, and BPA inhibited serum LH and T levels (124). Rats were exposed to 2.4 μg/kg BW/day BPA from GD12 to PND21, and BPA inhibited T levels in adulthood (124). Prepubertal mice were administered BPA via gavage for 56 days, and they had significantly lower free T levels without a change in LH levels (126). Prepubertal Wistar male rats (28 days old) were injected subcutaneously with 20, 100, and 200 mg/kg BW/day BPA, and BPA inhibited plasma T and LH for 6 weeks but did not affect FSH levels. BPA down-regulated steroidogenic enzymes and cholesterol carrier proteins in LCs and decreased LC number (127) (Table 2).

### Effects of Adult BPA Exposure on Male Reproduction

Adult male Swiss mice were given BPA by gavage of 5, 25, and 100 μg/kg BW for 28 days. BPA significantly lowered absolute testis weights, seminal vesicle weight, and sperm count and fertility rate (125). Adult male rats were exposed subcutaneously to 1 mg/rat BPA for 14 days. BPA decreased plasma T level and increased LH levels, suggesting that BPA directly inhibits LC function (128). Adult rats were administered via gavage of 10 mg/kg BW/day BPA for 14 days. BPA lowered T levels, decreased testis weight, and inhibited antioxidants, and co-treatment with an antioxidant (lipoic acid) could reverse it (112). Adult male rats were administered by gavage of 0.005, 0.5, 50, and 500 μg/kg BW/day BPA for 45 days. BPA significantly decreased insulin, insulin receptor, insulin receptor substrate-1, phosphoinositide 3-kinase (PI3K), and GLUT-2 in the testis as well as HSD3B1, HSD17B3, and StAR protein levels and T levels (131). Adult male rats were gavaged with 400 or 800 μmol/kg BW/day BPA for 14 days. BPA significantly decreased CYP17A1, POR, CYP1B1, and CYP2A1 protein levels without affecting HSD3B1 protein levels (132) and this potency of BPA was similar to 4 μmol/kg BW/day E_2_ (132). Treatment of ALCs with 0.01 μM BPA decreased T synthesis by down-regulating expression of *Cyp17a1* (124) (Table 2). This further demonstrates that BPA has different effects depending on doses.

## Human Studies

### Human Epidemiological Study

Some epidemiological studies have explored the relationship between human exposure to BPA during pregnancy and male reproductive diseases. The results are contradictory. Fénichel et al. measured unconjugated BPA levels in cord blood in 152 boys born after GW34 with cryptorchidism and 106 controls and did not find any association between BPA and cryptorchidism (133). Cord blood BPA levels were measured in 52 neonates with cryptorchidism and 128 controls in France. No correlation was found between BPA and T or cryptorchidism, but a significant negative correlation was found between BPA and INSL3 (18). Because INSL3 and T are important for testis descent, no relationship of BPA with cryptorchidism might be involved in more confounding factors. Serum BPA levels were detected in 98 (1–4 years old) unilateral cryptorchidism boys and 57 controls. No association between free BPA levels and cryptorchidism was found. However, they did find a significant association between total BPA levels and cryptorchidism (134). Fernandez et al. measured free BPA levels in term placenta in 28 boys of cryptorchidism/hypospadias and 51 controls, finding an association between BPA levels and cryptorchidism/hypospadias in the third tertile of cases (135). Miao et al. investigated maternal occupational exposure to BPA and AGD in 56 BPA-exposed male offspring and 97 unexposed controls and found that BPA was significantly negatively correlated with AGD (136). Liu et al. investigated the effect of BPA on sex hormone levels in 100 mother–infant pairs in two hospitals in China and found that maternal urinary BPA levels were negatively correlated with male fetal cord blood T levels and T/E2 ratios in male fetal cord blood without association with AGD (137). Therefore, more human studies are needed to clarify the effect of BPA on FLC functions of male fetuses and newborns.

For BPA-mediated effects on adult reproduction, Adoamnei et al. measured urinary BPA levels, serum LH levels, and sperm counts in 215 healthy young men (ages 18–23 years) in southern Spain, and found that urinary BPA was positively associated with serum LH levels and negatively with sperm concentrations, suggesting that BPA disrupts LC function and spermatogenesis (138). Den Hond et al. measured the urinary BPA levels and serum sex hormones in 163 subfertile men in four fertility clinics and found that there was a negative association between urinary BPA concentrations and serum T levels (139). Meeker et al. measured urinary BPA levels and serum reproductive hormone levels in 167 infertile men and found an inverse relationship between urinary BPA levels and free T (T/SBBG) (140). Mendiola et al. reported on 375 men with partners of pregnant women in four cities of the United States and found that urinary BPA level was not associated with semen quality, but was negatively related to free T index and positively related to SHBG (141).

### *In vitro* Studies Using Human Testis

The effect of BPA on FLC function was evaluated in human fetal testes. Exposure of BPA to human GW6-11 fetal testis explants for 3 days did not affect T secretion at 1 nM, but significantly lowered T secretion at 10 and 10 μM (142). Ben Maamar et al. found that BPA exposure to human GW7-12 human fetal testis explants for 3 days significantly inhibited T synthesis under the basal and LH or hCG-stimulated conditions at 10 μM (143, 144). BPA exposure also inhibited T secretion under a basal condition at 10 nM, but not under a LH-stimulated condition at this low concentration (143). Similar data were observed on the basis of BPA exposure to GW6-11 human testis and LH-stimulated T synthesis (41). Interestingly, Eladak et al. performed the first and second trimester human fetal testis xenograft to explore effect of BPA on T secretion and found that exposure of host mice to 10 μM BPA in water or 0.5 or 50 μg/kg BPA via gavage for 35 days did not influence T secretion from xenografts (41, 145).

## BPA Analogs

### Exposure of BPA Analogs

Due to strict restrictions on the production and use of BPA, several BPA analogs are gradually replacing BPA. Recent studies have reported that there was widespread exposure to a variety of chemicals with structural or functional similarity to BPA, referred to as BPA analogs (Table 1). BPA and its analogs were reported to exist in food stuffs (16, 146) and indoor dust (147) in both China and the United States. BPS and BPF are highly detectable in many water supply systems (148) and paper (149). BPA analogs can enter human tissues, circulation, and urine. In a survey for 190 women in Hangzhou, China, showed that, besides BPA (average level of 2.5 ng/mL), BPS (0.19 ng/mL) and BPAF (0.092 ng/mL) were also detectable in breast milk (150). In the serum samples of 181 Chinese pregnant women, BPS, BPF, BPAF, BPB, BPP, BPZ, BPAP, TBBPA, tetrabromobisphenol S (TBBPS), and tetrachlorobisphenol A (TCBPA) were detected, and TBBPS was 0.593 ng/mL and BPS was 0.113 ng/mL (151). BPB was detected in the urine of Portuguese volunteers, and its level was similar to BPA (152) (Table 1).

### *In vitro* Studies of BPA Analogs

Despite extensive research on the effects and toxicity of BPA on the male reproductive endocrine system in mammals, including humans, little is known about the activity of most BPA analogs. Several studies have been conducted on the toxicological effects of certain BPA analogs on Leydig cell function.

As mentioned above, LCs contain NR3C4 and androgen agonists, and antagonists can affect their development and function. The effects of BPA, BPF, BPS, and tetrabromobisphenol (TBBPA) on the activation of human NR3C4 were studied *in vitro*. BPA, BPF, and TBBPA antagonized NR3C4 activation with IC_50_ values of 39, 20, and 0.982 μM, while BPS did not affect it (153) (Table 3). Using a human recombinant androgen receptor (NR3C4) competitive binding test, it was found that BPB binds NR3C4 at a potency similar to BPA (157, 158). However, BPS bound NR3C4 weakly (157). BPA and its analogs were compared using *in vitro* and *in vivo* reporter assays for androgen agonism and antagonism. BPA significantly antagonized DHT androgenic activity in mouse fibroblast cell line NIH3T3 with TMBPA> BPAF >BPAD >BPB >BPA, whereas TBBPA and TCBPA were inactive (159). In another assay, like BPA, the following BPA analogs, TBBPA, BPAF, BPB, BPZ, BPE, 4,4-BPF, 2,2-BPF, BPC, TGSA, and TMBPA were NR3C4 antagonists between 3 and 100 μM, where BPS and TCBPA were inactive (160).

**Table 3:** Bisphenols as estrogen receptor agonists and androgen receptor (NR3C4) antagonists.

	**ESR1 agonist**						**ESR2 agonist**				**NR3C4 antagonist**	
**Chemical**	**EC_**50**_ (nM)^**a**^**	**Rel to BPA**	**EC_**50**_ (nM)^**b**^**	**Rel to BPA**	**EC_**50**_ (nM)^**c**^**	**Rel to BPA**	**EC_**50**_ (nM)^**a**^**		**EC_**50**_ (nM)^**c**^**	**Rel to BPA**	**EC_**50**_ (nM)^**c**^**	**Rel to BPA**
BPA	1200	1	1780	1	180	1	350	1	250	1	17500	1
T	IA	IA	IA	IA	IA	IA	IA	IA	IA	IA	2.8	6250
E2	0.042	28571	0.88	2225	0.9	200	1.1	318	0.3	833	30	583
BPAF	130	9	53.4	33.3	ND	ND	46	7.6	ND	ND	ND	ND
BPAP	-	-	259	6.9	2600	0.07	-	-	-	-	5400	3.2
BPB	320	4	195	9.1	ND	ND	-	-	ND	ND	ND	ND
BPC	780	2	2.81	633	3900	0.05	3200	9.1	-	-	1800	9.7
BPE	1400	1	ND	ND	ND	-	460	1.3	ND	ND	ND	ND
BPF	1600	1	ND	ND	1800	0.1	1300	3.7	3800	0.07	5800	3.0
BPFL	ND	ND	2230	0.8	-	-	ND		IA	IA	30	583
BPH	ND	ND	ND	ND	-	-	ND		-	-	1100	15.9
BPP	5600	0.2	176	10.1	ND	ND	-	-	ND	ND	ND	ND
BPS	1300	1	ND	ND	ND	ND	2100	6	ND	ND	ND	ND
BPZ	400	3	56.9	31.3	80	2.3	500	1.4	1100	0.23	1600	10.9
TDP	ND	ND	ND	ND	480	0.4	ND	ND	1100	0.23	5800	3.0
TMBPA	1100	1.1	1630	1.1	-	-	-	-	-	-	1100	15.9

a Potency of bisphenols in estrogen receptor (ESR) and androgen receptor (NR3C4) luciferase reporter gene assays (154);

b Ligand binding assay (155);

c *Ligand binding assay (156); IA, inactive; ND, not detected; -, no active activity; REL, potency relative to BPA*.

A series of estrogen receptor luciferase assays of BPA analogs in all 127 test compounds showed that BPC bound ESR1 with the highest affinity, with IC_50_ of 2.81 nM, and other BPA analogs such as BPAF (53.4 nM), BPM (56.8 nM), BPZ (56.9 nM), BPP (176 nM), BPB (195 nM), BPAP (259 nM), and BPA (1,780 nM) (155) (Table 3). Estrogen receptor binding experiments have shown similar effects of these BPA analogs (154, 156) (Table 3). Comparing the estrogen activity of BPA and its analogs in human breast cancer cell line MCF-7, the results showed that the estrogen activity was TCBPA> BPB> BPA> TMBPA (159). Using an *in vivo* uterotrophic assay in ovariectomized mice, anti-estrogenic activity against E_2_ was observed with TMBPA and TBBPA (159).

Compared with ESR1, BPAF also binds to ESR2 more effectively. The IC_50_ value of BPAF for ESR2 as an antagonist is 18.9 nM. Reporter gene assay showed that BPAF is a full agonist of ESR1, inactive to ESR2, and has very weak binding to ERR3 (161).

*In vitro* studies showed that after 24 h of treatment, BPAF was found to dose-dependently inhibit the production of P4 in mLTC-1 tumor LCs after 24 h of treatment with an IC_50_ value of 70.2 μM. BPAF also lowered intracellular cAMP levels and down-regulated *Scarb1* and *Cyp11a1* expression without affecting *Star* expression (162). This indicates that at high concentrations, BPAF has similar effect to BPA.

When MA-10 tumor LCs were treated with BPA analogs, TBBPA induced T synthesis, while BPF and BPS increased P4 levels (153). Fetal human testis was exposed to BPA, BPF, and BPS *in vitro*. These compounds inhibited T secretion at 10 nM (41). Fetal mouse testis was exposed to BPA and its analogs; these chemicals inhibited T secretion at higher concentrations, and the minimum effective concentrations were 1 μM for BPA and BPF as well as 100 nM for BPS (41). These data indicate that there is species-dependent difference for the inhibition of T synthesis between humans and mice, and human is more sensitive to BPA analogs than mouse. These chemicals also lowered *Insl3* transcription level at 10 μM in fetal mouse testis (41).

### *In vivo* Studies of BPA Analogs

Only some reproductive and developmental toxicity studies have been conducted on BPA analogs. BPAF did not change fetal T secretion from male fetuses on GD18 when exposed to BPAF by GD14 to 18 at a dose of 200–500 mg/kg/day (163). Exposure of rats to 5, 25, and 50 μg/L BPA and its analogs BPB, BPF, and BPS from GD1 to GD21 in drinking water caused significantly low antioxidant enzyme, plasma testosterone, and estrogen concentrations and altered morphological changes of testis and epididymis in male offspring after birth (164). *In vivo* studies of 5 mg/kg/day of BPA, BPB, BPF, and BPS exposed to adult male rats for 28 days showed that they led to decreased T levels and increased ROS levels (165). Male prepubertal rats exposed to 5, 25, and 50 μg/L BPA, BPB, BPF, and BPS in drinking water for 48 weeks also showed a decrease in T levels in the highest dose group (166). These results indicate that BPA analogs BPB, BPF, and BPS have similar effects on the development of the male reproductive system to BPA.

## Conclusion

BPA is a ubiquitous environmental pollutant, mainly from the manufacture and use of plastics and its degradation of waste related to industrial plastics. More and more animal experiments have shown that BPA has endocrine disruption to the development and function of LCs. Studies on laboratory animals have shown that the effect of BPA is usually more harmful in the uterus, which is a critical stage of embryonic development. BPA has been found to cause defects in the embryo, such as feminization of the male fetus, atrophy of the testes and epididymis, as well as shortened AGD and changes in adult sperm parameters. BPA also disrupts the development of LCs after birth and the function of LCs in adulthood. BPA may have several molecular mechanisms: (1) binding to different ESR (ERS1 and ERS2) and ERR (1-3) as agonists, and NR3C4 as antagonist (Figure 1); (2) binding to the membrane receptor (GPER) (Figure 1); (3) direct inhibition of steroidogenic enzyme activity; and (4) stimulation of ROS production. Epidemiological studies provide some data indicating that BPA can change male reproductive function in men. There are dose-dependent effects, including low-dose and high-dose effects and species-dependent effects. Human testes may be more sensitive to the T inhibition of BPA analogs.

## Author Contributions

XL and ZW wrote the paper. R-SG and YZ edited the paper. YW prepared the tables. JM draw the figure. All authors contributed to the article and approved the submitted version.

## Conflict of Interest

The authors declare that the research was conducted in the absence of any commercial or financial relationships that could be construed as a potential conflict of interest.
